# Supplementary material for: Interruption of post-Golgi STING trafficking activates tonic interferon signaling
Source: Nat Commun. 2022 Nov 15;13:6977. doi: 10.1038/s41467-022-33765-0 (PMC9666523; doi:10.1038/s41467-022-33765-0)
Supplement: Supplementary file 1 — Supplementary Information [file 41467_2022_33765_MOESM1_ESM.pdf]

SUPPLEMENTARY MATERIALS

Interruption of post-Golgi STING trafficking activates tonic interferon signaling

Xintao Tu[1], Ting-Ting Chu[1], Devon Jeltema[1], Kennady Abbott[1], Kun Yang[1], Cong Xing[1], Jie Han[1], Nicole Dobbs[1], and Nan Yan[1,2,*]

SUPPLEMENTARY FIGURES 1-10

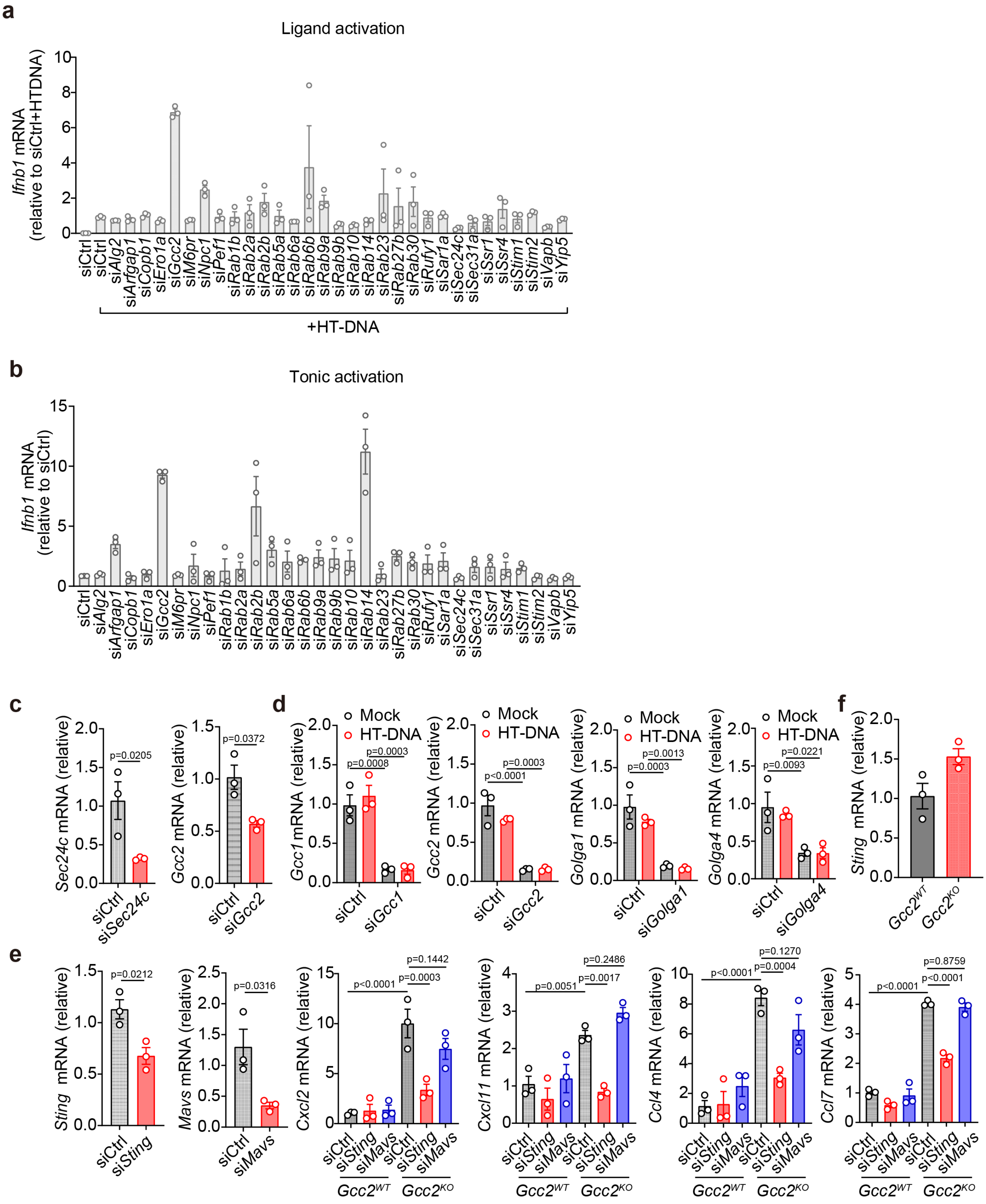
a
Ligand activation
Ifnb1 mRNA (relative to siCtrl+HTDNA)
siCtrl
siCtrl
siAlg2
siArfgap1
siCopb1
siEro1a
siGcc2
siM6pr
siNpc1
siPef1
siRab1b
siRab2a
siRab2b
siRab5a
siRab6a
siRab6b
siRab9a
siRab9b
siRab10
siRab14
siRab23
siRab27b
siRab30
siRufy1
siSar1a
siSec24c
siSec31a
siSsr1
siSsr4
siStim1
siStim2
siVapb
siYip5
+HT-DNA
b
Tonic activation
Ifnb1 mRNA (relative to siCtrl)
c
Sec24c mRNA (relative)
p=0.0205
siCtrl
siSec24c
Gcc2 mRNA (relative)
p=0.0372
siCtrl
siGcc2
d
Mock
HT-DNA
Gcc1 mRNA (relative)
p=0.0008
p=0.0003
siCtrl
siGcc1
Gcc2 mRNA (relative)
p<0.0001
p=0.0003
siCtrl
siGcc2
Golga1 mRNA (relative)
p=0.0003
p=0.0013
siCtrl
siGolga1
Golga4 mRNA (relative)
p=0.0093
p=0.0221
siCtrl
siGolga4
f
Sting mRNA (relative)
Gcc2^WT
Gcc2^KO
e
Sting mRNA (relative)
p=0.0212
siCtrl
siSting
Mavs mRNA (relative)
p=0.0316
siCtrl
siMavs
Cxcl2 mRNA (relative)
p<0.0001
p=0.0003
p=0.1442
Cxcl11 mRNA (relative)
p=0.0051
p=0.0017
p=0.2486
Ccl4 mRNA (relative)
p<0.0001
p=0.0004
p=0.1270
Ccl7 mRNA (relative)
p<0.0001
p<0.0001
p=0.8759
siCtrl
siSting
siMavs
Gcc2^WT
Gcc2^KO

Supplementary Figure 1. Secondary siRNA screen of selected STING cofactors identified from the primary proteomic screen.

a, b Ligand activation **a** and tonic activation **b** of *Ifnb1* mRNA expression. Wild-type MEFs were transfected with siRNA to knocking down each selected candidate cofactors. Then, knockdown cells were either simulated with HT-DNA (1 μg/mL, 4 h) then qRT-PCR for *Ifnb1* expression (A) or directly measured *Ifnb1* expression without stimulation **b**. Fold-changes were determined by normalizing to control siRNA in either assay.

c-e siRNA knockdown efficiency of *Sec24c* and *Gcc2* **c**, *Gcc1*, *Gcc2*, *Golga1* and *Golga4* **d**, *Sting* and *Mavs* **e** in MEFs measured by qRT-PCR. n=3.

f qRT-PCR measurement of *Sting* mRNA level.

Data are representative of at least three independent experiments. Data (**a**-**e**) are shown as mean ± s.e.m. P values are determined by unpaired two-tailed Student's *t*-test (**c** and **e**) and One-way ANOVA (**d** and **e**). $*p < 0.05$, $**p < 0.01$, $***p < 0.001$,$****p < 0.0001$. ns, not significant.

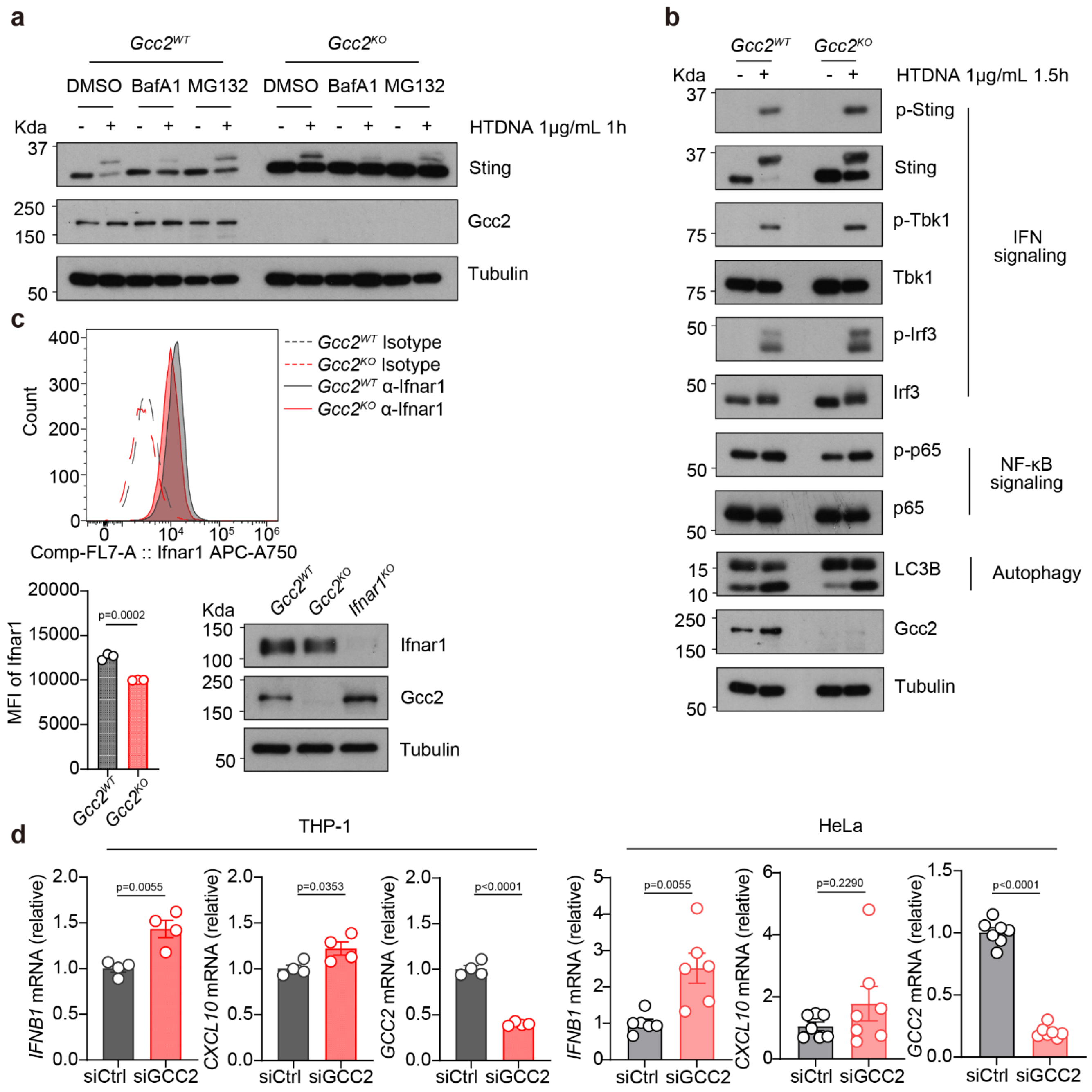

Supplementary Figure 2. STING lysosomal degradation, signaling activities in *Gcc2*^{KO} MEFs and *GCC2* knockdown human cells.

a Western blot analysis of STING degradation. *Gcc2*^{WT} and *Gcc2*^{KO} MEFs were pretreated with lysosome inhibitor Bafilomycin A1 or proteasome inhibitor MG132 prior to the HTDNA stimulation (1 μg/mL).

b Western blot analysis of STING signaling activities. *Gcc2*^{WT} and *Gcc2*^{KO} MEFs were stimulated with HTDNA (1 μg/mL) for 1.5 h. p-Sting, p-Tbk1 and p-Irf3 are key phosphorylation events of the STING-

dependent IFN signaling. p-p65 is a key phosphorylation event of the STING-dependent NF-kB signaling. LC3B conversion is key event of STING-dependent autophagy. n=3.

c Characterization of Ifnar1 expression in *Gcc2*^{WT} and *Gcc2*^{KO} MEFs. A representative histogram of cell surface Ifnar1 in *Gcc2*^{WT} and *Gcc2*^{KO} MEFs measured by FACS (top). A bar graph showing the mean fluorescence intensity of Ifnar1 (bottom left). Total Ifnar1 expression in whole cell lysate is determined by Western blot.

d qRT-PCR analysis of tonic *IFNB1* and *CXCL10* mRNA expression in THP-1 (n=4) and HeLa cells (n=6) after siRNA knockdown of GCC2 for 48 h.

Data are representative of at least three independent experiments. Around 30 cells in two-three different views were analyzed (**a-c**). Data (**d**) are shown as mean ± s.e.m. P values are determined by unpaired two-tailed Student's *t*-test in **c**,**d**. $*p < 0.05$, $**p < 0.01$, $***p < 0.001$ and $****p < 0.0001$. ns, not significant.

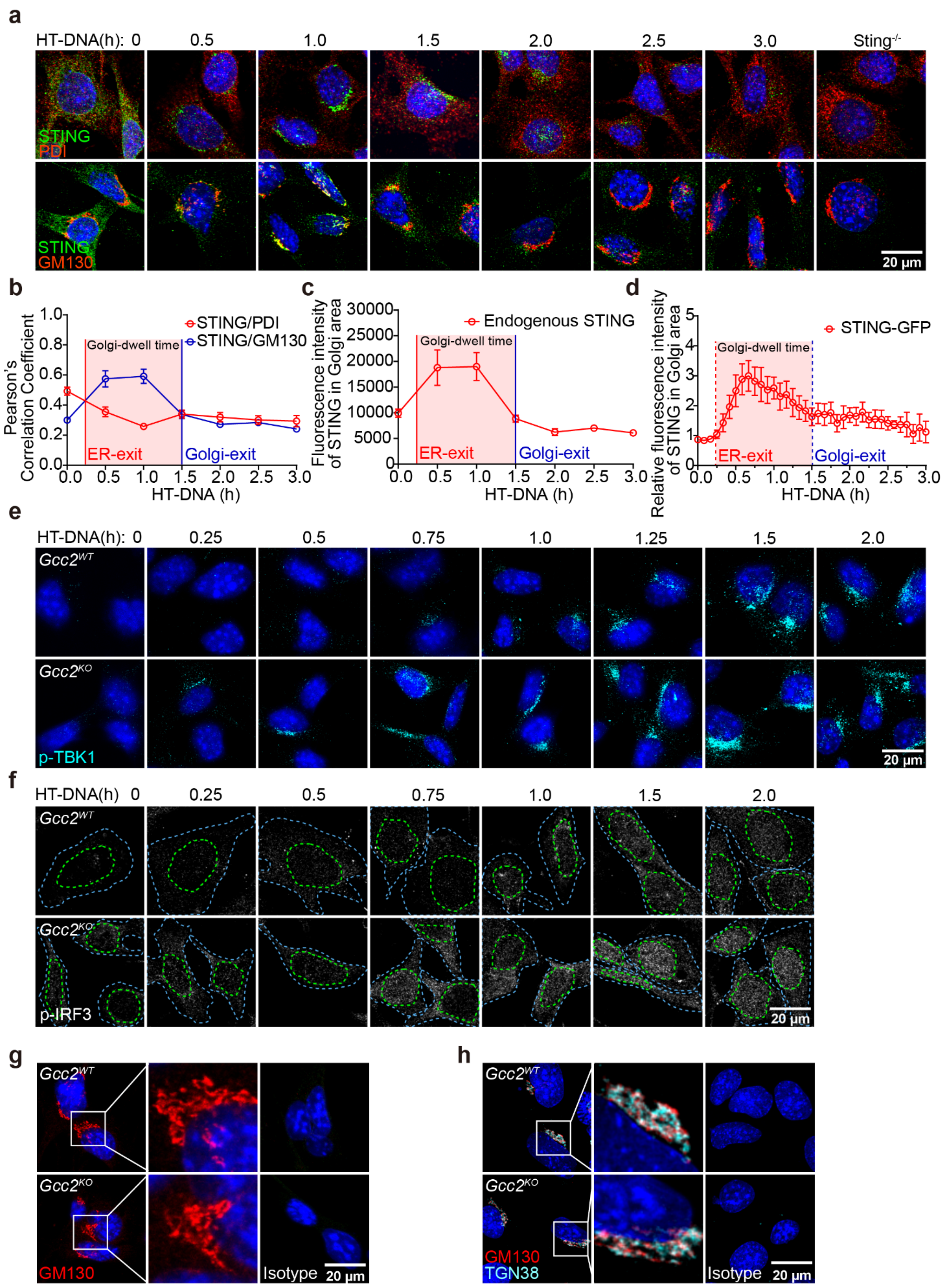

a
HT-DNA(h): 0
0.5
1.0
1.5
2.0
2.5
3.0
Sting-/-
STING
PDI
STING
GM130
20 μm
b
Pearson's Correlation Coefficient
STING/PDI
STING/GM130
Golgi-dwell time
ER-exit
Golgi-exit
HT-DNA (h)
c
Fluorescence intensity of STING in Golgi area
Endogenous STING
Golgi-dwell time
ER-exit
Golgi-exit
HT-DNA (h)
d
Relative fluorescence intensity of STING in Golgi area
STING-GFP
Golgi-dwell time
ER-exit
Golgi-exit
HT-DNA (h)
e
HT-DNA(h): 0
0.25
0.5
0.75
1.0
1.25
1.5
2.0
Gcc2WT
Gcc2KO
p-TBK1
20 μm
f
HT-DNA(h) 0
0.25
0.5
0.75
1.0
1.5
2.0
Gcc2WT
Gcc2KO
p-IRF3
20 μm
g
Gcc2WT
Gcc2KO
GM130
Isotype
20 μm
h
Gcc2WT
Gcc2KO
GM130
TGN38
Isotype
20 μm

Supplementary Figure 3. A quantitative time-lapse microscopy assay for STING trafficking and signaling.

a Confocal microscopy images showing endogenous STING trafficking in wild-type MEFs followed by HT-DNA stimulation (1 μg/mL) for indicated times (top, same below). *Sting*$^{-/-}$ MEFs (panels on the right) were used to ensure specificity of the STING antibody staining. Endogenous STING in green, PDI (an ER marker) or GM130 (a Golgi marker) in red, DAPI in blue. Scale bar, 20μm. n=3.

b Quantification of endogenous STING colocalization with the ER or the Golgi during trafficking. Two-color images were analyzed by Fiji to determine Pearson's Correlation Coefficient shown as a function of time. Highlighted area indicated STING Golgi-dwell time. The colored area corresponds to Golgi residency. Same below. n=3.

c Quantification of fluorescence intensity of endogenous STING in the Golgi area (GM130^{+} area) as a function of time. Highlighted area indicated STING Golgi-dwell time. n=3.

d Quantification of fluorescence intensity of STING-GFP in live cells as a function of time. Highlighted area indicated STING Golgi-dwell time. ER-exit (dotted line) was estimated by increase of GFP signal and Golgi-exit (dotted line) was estimated by decrease of GFP signal. n=3.

e Representative images showing endogenous p-TBK1 staining in *Gcc2*^{WT} and *Gcc2*^{KO} MEFs after HT-DNA stimulation (1 μg/mL). Endogenous p-TBK1 in cyan and DAPI in blue. Scale bar, 20μm.

f Representative images showing endogenous p-IRF3 staining in *Gcc2*^{WT} and *Gcc2*^{KO} MEFs HT-DNA stimulation (1 μg/mL). Blue dotted lines indicate cell periphery. Green dotted lines indicate the nucleus. Endogenous p-IRF3 in white. Scale bar, 20μm. n=3.

g, h Representative images showing normal Golgi morphology in *Gcc2*^{WT} and *Gcc2*^{KO} MEFs. Confocal microscopy images showing GM130, and TGN38 in *Gcc2*^{WT} and *Gcc2*^{KO} MEFs at the resting state. Cis-Golgi marker GM130 in Red, trans-Golgi marker TGN38 in Cyan. Scale bar, 20μm. n=3.

Data are representative of at least three independent experiments. Around 10 cells in two different views were analyzed (**b-d**). Data (**b-d**) are shown as mean ± s.e.m.

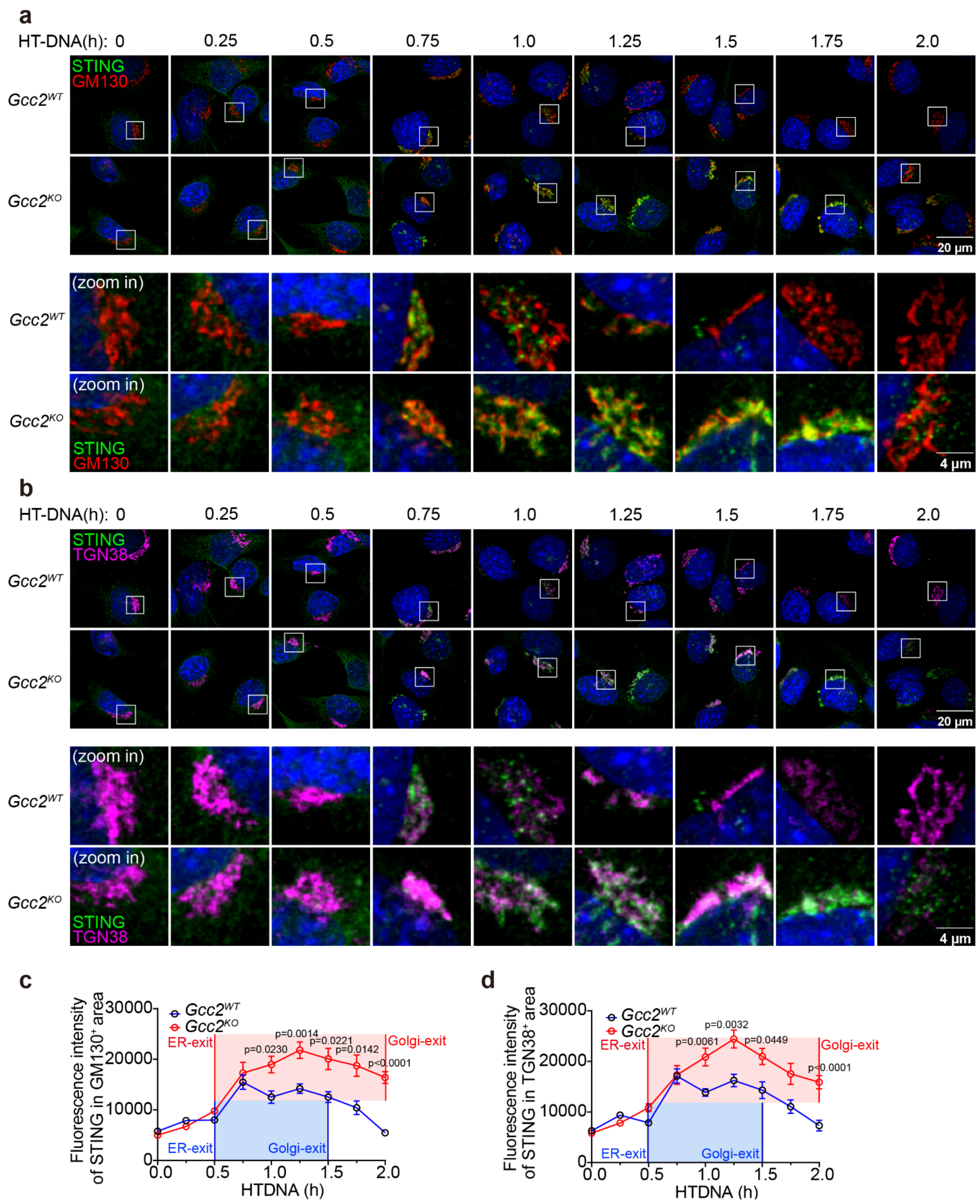
a
HT-DNA(h): 0
0.25
0.5
0.75
1.0
1.25
1.5
1.75
2.0
STING
GM130
Gcc2WT
Gcc2KO
20 µm
(zoom in)
4 µm
b
TGN38
c
d
Fluorescence intensity of STING in GM130+ area
Fluorescence intensity of STING in TGN38+ area
HTDNA (h)
ER-exit
Golgi-exit
p=0.0230
p=0.0014
p=0.0221
p=0.0142
p<0.0001
p=0.0061
p=0.0032
p=0.0449

Supplementary Figure 4. STING Golgi-dwell time measurement using cis- and trans-Golgi markers.

a, b Confocal microscopy images showing endogenous STING trafficking in *Gcc2*^{WT} and *Gcc2*^{KO} MEFs followed by HT-DNA stimulation (1 µg/mL) for indicated times (top, same below). Endogenous STING in green, GM130 (a cis-Golgi marker) in red **a**, TGN38 (a trans-Golgi marker) in magenta **b,** DAPI in blue. A white box indicated enlarged area shown in the zoom-in panel below. Scale bar of the original images and zoom in images are 20µm and 4µm, respectively. n=3.

c Quantification of fluorescence intensity of endogenous STING in the cis-Golgi (GM130^{+} area) using Fiji. Asterisk indicates significant differences. The colored area corresponds to Golgi residency. Same below. n=3.

d Quantification of fluorescence intensity of endogenous STING in the trans-Golgi (TGN38^{+} area) using Fiji. Asterisk indicates significant differences. n=3.

Data are representative of at least three independent experiments. At least 30 cells in two-three different views were analyzed (**a, b**). Data (**c, d**) are shown as mean ± s.e.m. P values are determined by Two-way ANOVA in **c,d**. $*p < 0.05$, $**p < 0.01$, $***p < 0.001$ and $****p < 0.0001$.

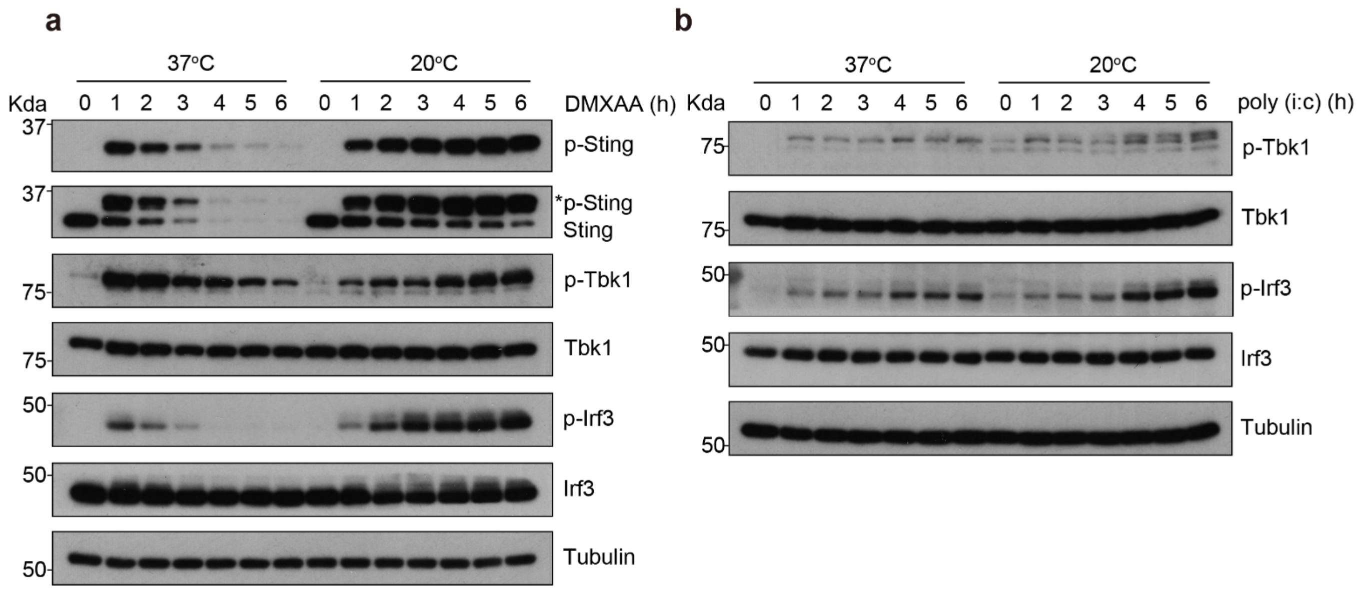

Supplementary Figure 5. STING signaling is selectively affected by global arrest of post-Golgi trafficking.

a Western blot analysis of STING signaling kinetics at different temperatures. *Gcc2*^{WT} and *Gcc2*^{KO} MEFs were first acclimated in 37°C or 20°C then stimulated with cell-permeable STING agonist DMXAA (10 μg/mL) for indicated times while remaining at the same temperature.

b Western blot analysis of dsRNA-induced IFN signaling at different temperatures. *Gcc2*^{WT} and *Gcc2*^{KO} MEFs were first acclimated in 37°C or 20°C then stimulated with synthetic dsRNA poly (I:C) (1 μg/mL) for indicated times while remaining at the same temperature.

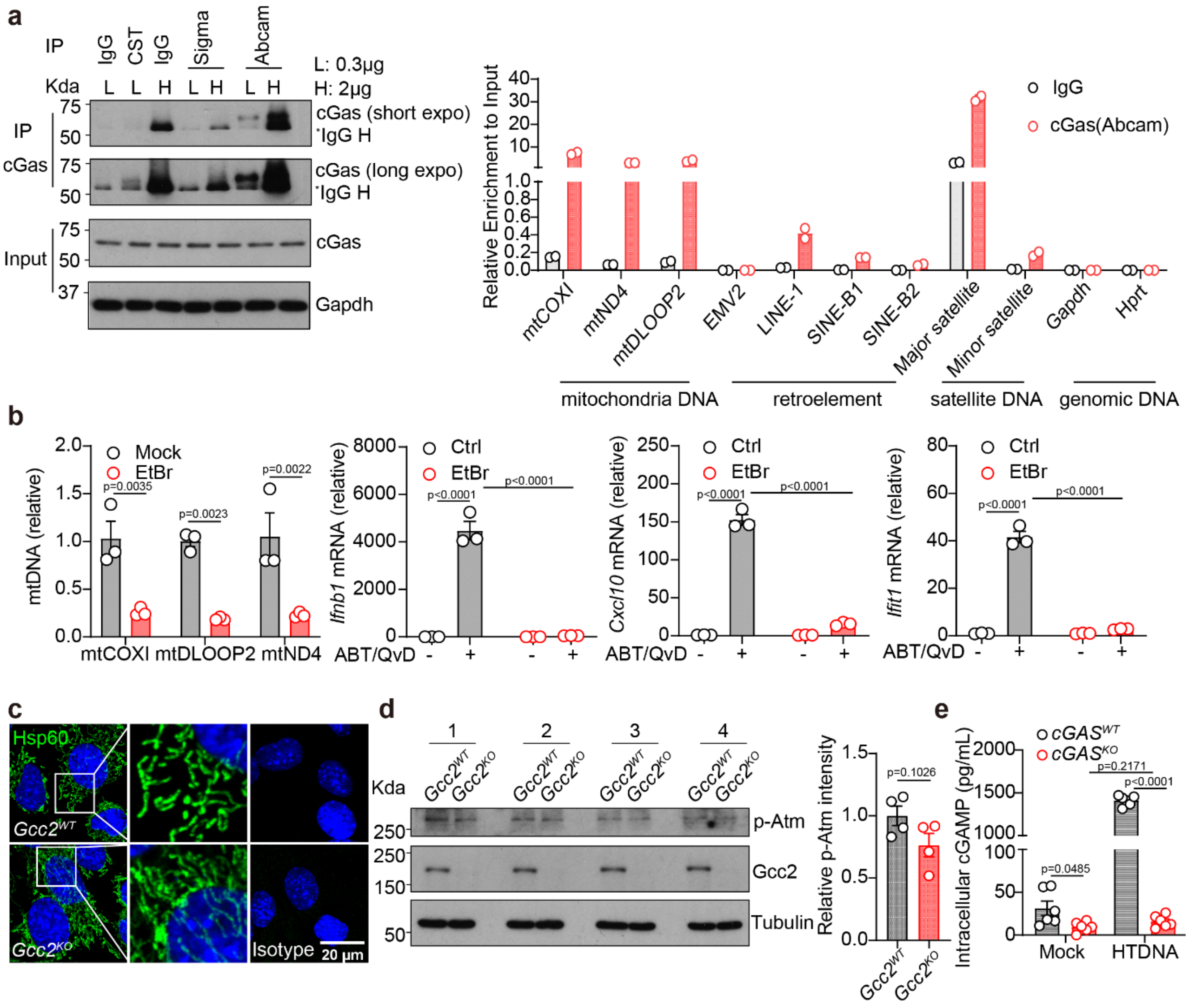

Supplementary Figure 6. Cellular sources of DNA for cGAS.

a Endogenous cGAS IP and DNA analysis. Wildtype MEFs whole cell lysate was used for IP with control IgG or α-cGAS antibody from indicated vendors (Sigma or Abcam) at two different concentrations (L, 0.3 μg; H, 2 μg). Abcam α-cGAS antibody at 0.3 μg showed the best enrichment with minimal background and was used for DNA analysis. cGAS-bound DNA species were analyzed by qPCR after IP (right). n=2.

b mtDNA was depletion by EtBr treatment and IFN signaling. qPCR analysis of mtDNA content in EtBr-treated cells (left). qPCR analysis of IFN signaling following ABT/QvD stimulation in control and EtBr-treated cells (right). n=3.

c Representative images showing normal mitochondria morphology in *Gcc2*^{WT} and *Gcc2*^{KO} MEFs.

d Western blot analysis of DNA damage marker p-ATM in *Gcc2*^{WT} and *Gcc2*^{KO} MEFs. Samples from four different experiments were examined in the same SDS-PAGE gel. Quantification of p-ATM intensity was shown as a bar graph on the right. n=4.

e ELISA analysis of intracellular 2'3'-cGAMP in *cGAS*^{WT} and *cGAS*^{KO} MEFs with or without HTDNA stimulation. n=6.

Data are representative of at least three independent experiments. Data (**a, b, e**) are shown as mean ± s.e.m. P values are determined by One-way ANOVA (**b** and **e**), unpaired two-tailed Student's *t*-test in (**d**). $*p < 0.05$, $**p < 0.01$, $***p < 0.001$, $****p < 0.0001$. ns, not significant.

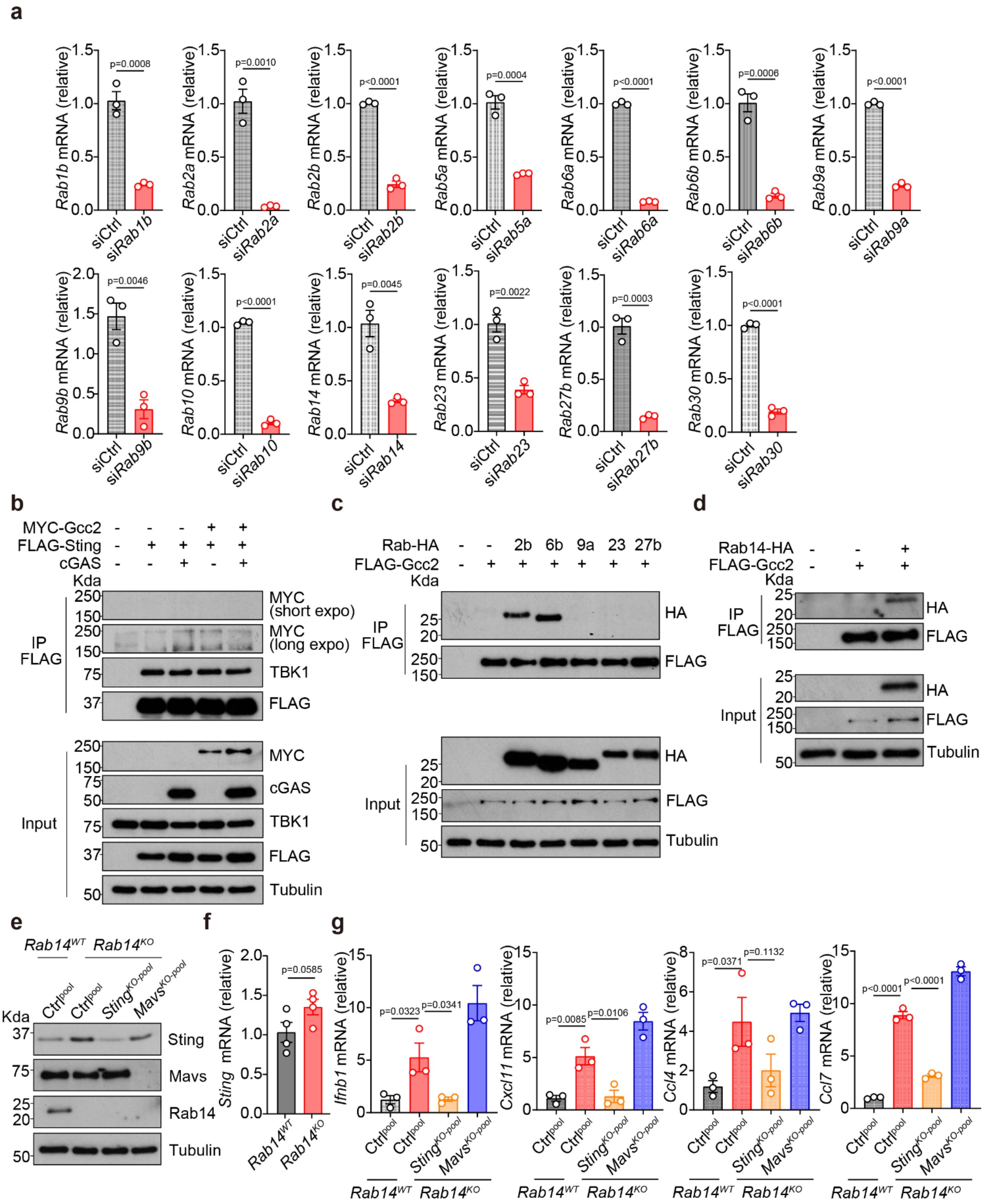
a
Rab1b mRNA (relative)
siCtrl
siRab1b
p=0.0008
Rab2a mRNA (relative)
siRab2a
p=0.0010
Rab2b mRNA (relative)
siRab2b
p<0.0001
Rab5a mRNA (relative)
siRab5a
p=0.0004
Rab6a mRNA (relative)
siRab6a
p<0.0001
Rab6b mRNA (relative)
siRab6b
p=0.0006
Rab9a mRNA (relative)
siRab9a
p<0.0001
Rab9b mRNA (relative)
siRab9b
p=0.0046
Rab10 mRNA (relative)
siRab10
p<0.0001
Rab14 mRNA (relative)
siRab14
p=0.0045
Rab23 mRNA (relative)
siRab23
p=0.0022
Rab27b mRNA (relative)
siRab27b
p=0.0003
Rab30 mRNA (relative)
siRab30
p<0.0001
b
MYC-Gcc2
FLAG-Sting
cGAS
Kda
IP FLAG
MYC (short expo)
MYC (long expo)
TBK1
FLAG
Input
MYC
cGAS
TBK1
FLAG
Tubulin
c
Rab-HA
FLAG-Gcc2
Kda
2b
6b
9a
23
27b
IP FLAG
HA
FLAG
Input
HA
FLAG
Tubulin
d
Rab14-HA
FLAG-Gcc2
Kda
IP FLAG
HA
FLAG
Input
HA
FLAG
Tubulin
e
Rab14WT
Rab14KO
Kda
Ctrlpool
Ctrlpool
StingKO-pool
MavsKO-pool
Sting
Mavs
Rab14
Tubulin
f
Sting mRNA (relative)
p=0.0585
Rab14WT
Rab14KO
g
Ifnb1 mRNA (relative)
p=0.0323
p=0.0341
Cxcl11 mRNA (relative)
p=0.0085
p=0.0106
Ccl4 mRNA (relative)
p=0.0371
p=0.1132
Ccl7 mRNA (relative)
p<0.0001
p<0.0001
Ctrlpool
Ctrlpool
StingKO-pool
MavsKO-pool
Rab14WT
Rab14KO

Supplementary Figure 7. Biochemical analysis of GCC2, Rab GTPases and STING protein interaction.

a siRNA knockdown efficiency of several Rab GTPases in MEFs measured by qRT-PCR. n=3.

b Co-immunoprecipitation analysis of Myc-GCC2 and FLAG-STING interaction. HEK293T cells were transfected with indicated plasmids (top) followed by IP with FLAG antibody. No interaction was detected.

c, d Co-immunoprecipitation analysis of FLAG-Gcc2 and various HA-Rab GTPases interaction. HEK293T cells were transfected with indicated plasmids (top) followed by IP with FLAG antibody. GCC2 interaction with Rab2a, Rab2b and Rab14 was detected.

e Western blot analysis of Rab, STING and MAVS protein knockout efficiency in indicated single and double knockout MEFs (top).

f qRT-PCR analysis of *Sting* mRNA expression in $Rab14^{WT}$ and $Rab14^{KO}$ MEFs. n=4.

g qRT-PCR analysis of *Ifnb1* and ISGs mRNA expression in $Rab14^{WT}$, $Rab14^{KO}$, $Rab14^{KO}Sting^{KO\text{-}pool}$, $Rab14^{KO}Mavs^{KO\text{-}pool}$ MEFs. n=3.

Data are representative of at least three independent experiments. Data (**a** and **f**) are shown as mean ± s.e.m. P values are determined by unpaired two-tailed Student's *t*-test in (**a** and **f**) and One-way ANOVA (**g**). $*p < 0.05$, $**p < 0.01$, $***p < 0.001$, $****p < 0.0001$. ns, not significant.

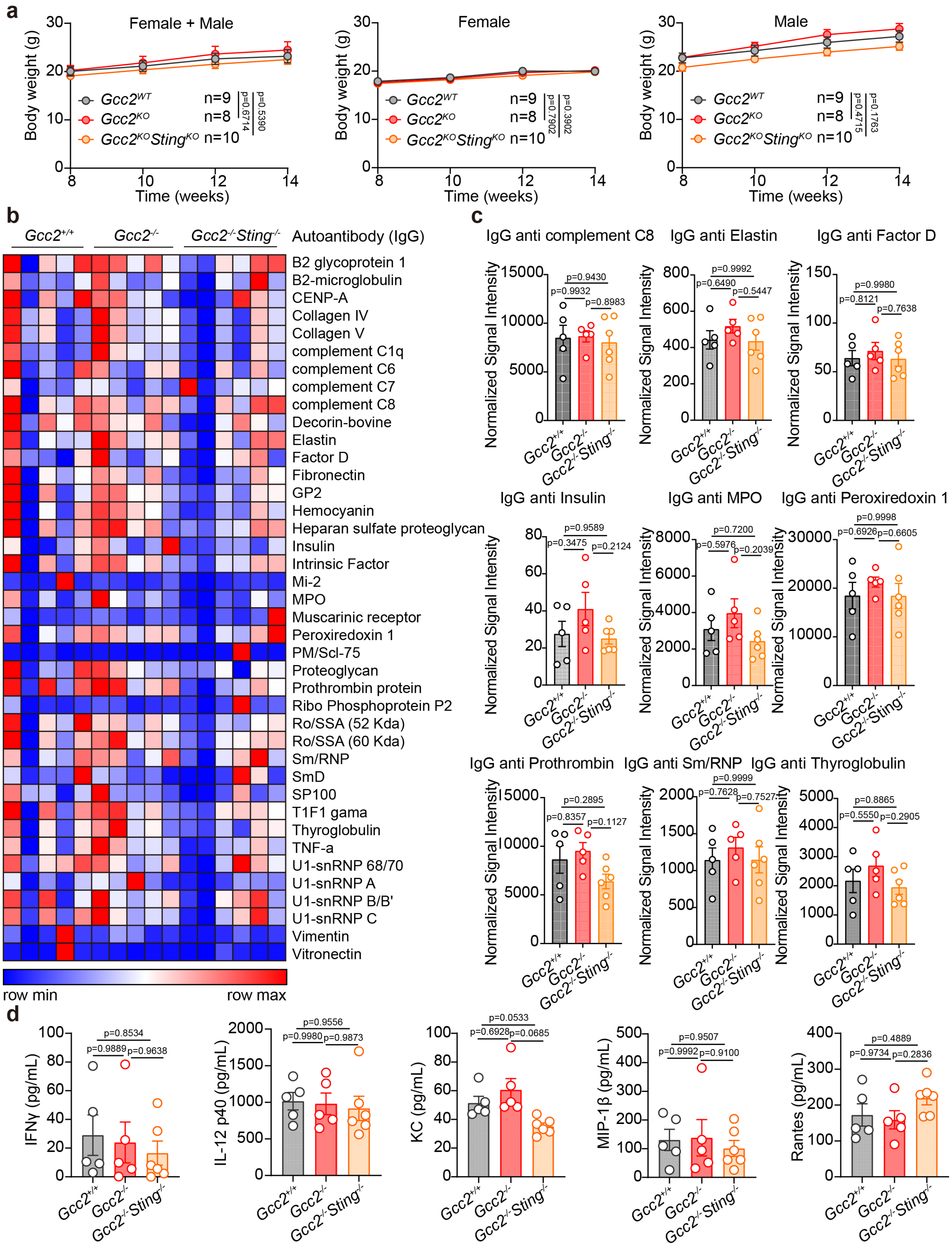

a
Female + Male
Female
Male
Body weight (g)
Time (weeks)
Gcc2^WT n=9
Gcc2^KO n=8
Gcc2^KO Sting^KO n=10
p=0.6714
p=0.5390
p=0.7902
p=0.3902
p=0.4715
p=0.1763
b
Gcc2+/+
Gcc2-/-
Gcc2-/-Sting-/-
Autoantibody (IgG)
B2 glycoprotein 1
B2-microglobulin
CENP-A
Collagen IV
Collagen V
complement C1q
complement C6
complement C7
complement C8
Decorin-bovine
Elastin
Factor D
Fibronectin
GP2
Hemocyanin
Heparan sulfate proteoglycan
Insulin
Intrinsic Factor
Mi-2
MPO
Muscarinic receptor
Peroxiredoxin 1
PM/Scl-75
Proteoglycan
Prothrombin protein
Ribo Phosphoprotein P2
Ro/SSA (52 Kda)
Ro/SSA (60 Kda)
Sm/RNP
SmD
SP100
T1F1 gama
Thyroglobulin
TNF-a
U1-snRNP 68/70
U1-snRNP A
U1-snRNP B/B'
U1-snRNP C
Vimentin
Vitronectin
row min
row max
c
IgG anti complement C8
IgG anti Elastin
IgG anti Factor D
IgG anti Insulin
IgG anti MPO
IgG anti Peroxiredoxin 1
IgG anti Prothrombin
IgG anti Sm/RNP
IgG anti Thyroglobulin
Normalized Signal Intensity
p=0.9430
p=0.9932
p=0.8983
p=0.9992
p=0.6490
p=0.5447
p=0.9980
p=0.8121
p=0.7638
p=0.9589
p=0.3475
p=0.2124
p=0.7200
p=0.5976
p=0.2039
p=0.9998
p=0.6926
p=0.6605
p=0.2895
p=0.8357
p=0.1127
p=0.9999
p=0.7628
p=0.7527
p=0.8865
p=0.5550
p=0.2905
Gcc2+/+
Gcc2-/-
Gcc2-/-Sting-/-
d
IFNγ (pg/mL)
IL-12 p40 (pg/mL)
KC (pg/mL)
MIP-1β (pg/mL)
Rantes (pg/mL)
p=0.8534
p=0.9889
p=0.9638
p=0.9556
p=0.9980
p=0.9873
p=0.0533
p=0.6928
p=0.0685
p=0.9507
p=0.9992
p=0.9100
p=0.4889
p=0.9734
p=0.2836

Supplementary Figure 8. Normal IgG autoantibodies, cytokines, and body weight in *Gcc2$^{-/-}$* mice.

a Body weight of *Gcc2$^{+/+}$* (n=9), *Gcc2$^{-/-}$* (n=8), *Gcc2$^{-/-}$Sting$^{-/-}$* (n=10) mice.

b A heatmap showing IgG autoantibody array analysis of *Gcc2$^{+/+}$*, *Gcc2$^{-/-}$*, *Gcc2$^{-/-}$Sting$^{-/-}$* mouse serum (6-month-old). n=5.

c Representative bar graphs of individual IgG autoantibody from **b**. n=5.

d Representative bar graphs of serum cytokines from *Gcc2$^{+/+}$*, *Gcc2$^{-/-}$*, *Gcc2$^{-/-}$Sting$^{-/-}$* mouse serum (6-month-old). n=5.

Data are representative of at least three independent experiments. Data are shown as mean ± s.e.m. P values are determined by One-way ANOVA in (**b** and **c**) or Two-way ANOVA in **a**. ns, not significant.

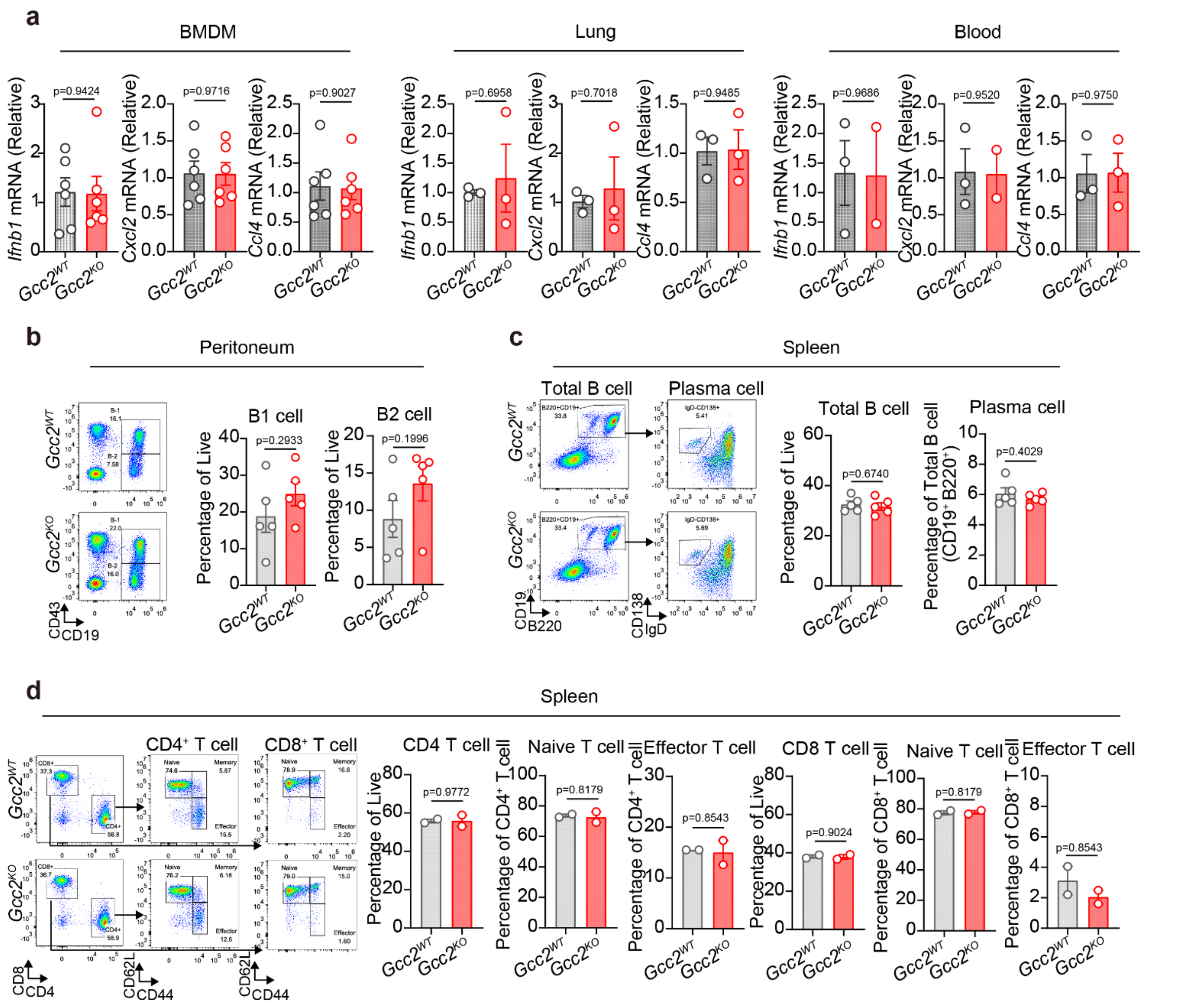

Supplementary Figure 9. Normal immune cell development and ISGs in *Gcc2*$^{-/-}$ mice.

a qRT-PCR analysis of *Ifnb1* and ISGs mRNA expression in BMDM, lung, and whole blood from *Gcc2*$^{+/+}$ and *Gcc2*$^{-/-}$ mice. n=6.

b Representative FACS plot of peritoneal B1/B2 cells from *Gcc2*$^{+/+}$ and *Gcc2*$^{-/-}$ mice. n=5.

c Representative FACS plot of splenic B cell populations from *Gcc2*$^{+/+}$ and *Gcc2*$^{-/-}$ mice. n=5.

d Representative FACS plot of splenic T cell populations from *Gcc2*$^{+/+}$ and *Gcc2*$^{-/-}$ mice. n=2.

Data are representative of at least three independent experiments. Data are shown as mean ± s.e.m. P values are determined by unpaired two-tailed Student's *t*-test. ns, not significant.

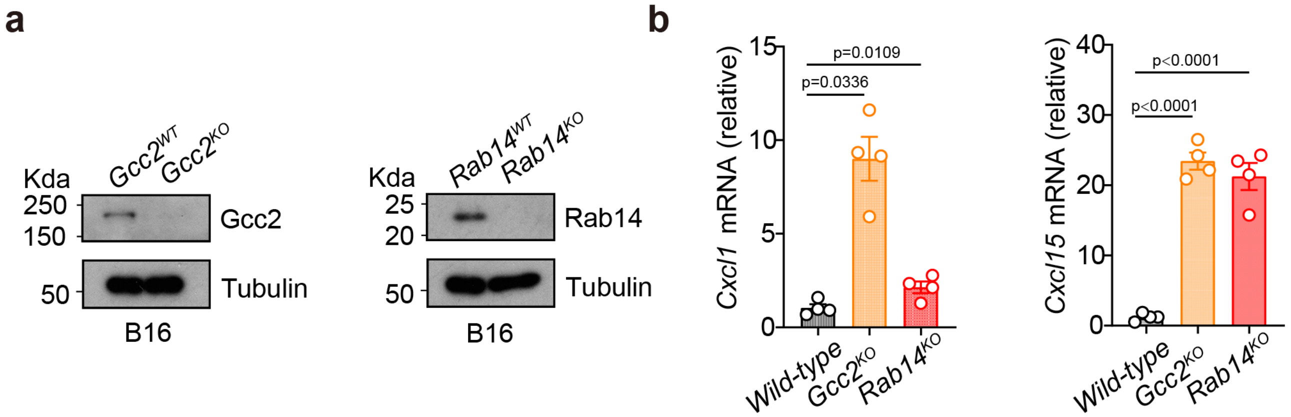

Supplementary Figure 10. *Gcc2* or *Rab14* knockout in B16 cells increase resting-state ISG expression.

a Western blot analysis of wild-type, $Gcc2^{KO}$ and $Rab14^{KO}$ B16 cells to confirm protein knockout.

b qRT-PCR analysis of ISG mRNA expression in wild-type, $Gcc2^{KO}$ or $Rab14^{KO}$ B16 cells. n=4.

Data are representative of at least three independent experiments. Data (**b**) are shown as mean ± s.e.m. P values are determined by One-way ANOVA. $*p < 0.05$, $**p < 0.01$, $***p < 0.001$ and $****p < 0.0001$.
