# Supplementary material for: Interruption of post-Golgi STING trafficking activates tonic interferon signaling
Source: Nat Commun. 2022 Nov 15;13:6977. doi: 10.1038/s41467-022-33765-0 (PMC9666523; doi:10.1038/s41467-022-33765-0)
Supplement: Supplementary file 3 — Reporting Summary [file 41467_2022_33765_MOESM3_ESM.pdf]

nature portfolio

Corresponding author(s): Nan Yan

Last updated by author(s): Sep 6, 2022

Reporting Summary

Nature Portfolio wishes to improve the reproducibility of the work that we publish. This form provides structure for consistency and transparency in reporting. For further information on Nature Portfolio policies, see our Editorial Policies and the Editorial Policy Checklist.

Statistics

For all statistical analyses, confirm that the following items are present in the figure legend, table legend, main text, or Methods section.

n/a	Confirmed	
☐	☒	The exact sample size (n) for each experimental group/condition, given as a discrete number and unit of measurement
☐	☒	A statement on whether measurements were taken from distinct samples or whether the same sample was measured repeatedly
☐	☒	The statistical test(s) used AND whether they are one- or two-sided<br>*Only common tests should be described solely by name; describe more complex techniques in the Methods section.*
☒	☐	A description of all covariates tested
☒	☐	A description of any assumptions or corrections, such as tests of normality and adjustment for multiple comparisons
☐	☒	A full description of the statistical parameters including central tendency (e.g. means) or other basic estimates (e.g. regression coefficient) AND variation (e.g. standard deviation) or associated estimates of uncertainty (e.g. confidence intervals)
☐	☒	For null hypothesis testing, the test statistic (e.g. F, t, r) with confidence intervals, effect sizes, degrees of freedom and P value noted<br>*Give P values as exact values whenever suitable.*
☒	☐	For Bayesian analysis, information on the choice of priors and Markov chain Monte Carlo settings
☒	☐	For hierarchical and complex designs, identification of the appropriate level for tests and full reporting of outcomes
☒	☐	Estimates of effect sizes (e.g. Cohen's d, Pearson's r), indicating how they were calculated

Our web collection on statistics for biologists contains articles on many of the points above.

Software and code

Policy information about availability of computer code

Data collection	Bio-Rad CFX Maestro, Zeiss Zen Blue, Canon scanner LIDE, BioTek Gen 5, BD FACStation
Data analysis	Prism 9, Image J 1.52, Autoquant X, Excel 2016, Synthego ICE2, Flowjo X

For manuscripts utilizing custom algorithms or software that are central to the research but not yet described in published literature, software must be made available to editors and reviewers. We strongly encourage code deposition in a community repository (e.g. GitHub). See the Nature Portfolio guidelines for submitting code & software for further information.

Data

Policy information about availability of data

All manuscripts must include a data availability statement. This statement should provide the following information, where applicable:

- Accession codes, unique identifiers, or web links for publicly available datasets
- A description of any restrictions on data availability
- For clinical datasets or third party data, please ensure that the statement adheres to our policy

Data are available from the corresponding author upon reasonable request

Field-specific reporting

Life sciences study design

All studies must disclose on these points even when the disclosure is negative.

Sample size	No sample size calculation was performed as no information on the experimental outcome was available at the time when this study was designed. Group sizes of more than 10 mice were chosen for experiments with 2-3 times replications based on previous published studies (Chu et al 2021 Nature) showing that these setups were sufficiently large to identify differences between groups.
Data exclusions	No data was excluded.
Replication	All experiments were replicated several times as indicated in the figure legends. All attempts of replication were successful.
Randomization	Mice were randomly allocated for each experiment. All animals were kept in a pathogen-free barrier facility at UT Southwestern Medical Center. For in vitro experiments, cells were also randomly allocated for each treatment condition.
Blinding	Investigators were not blinded to genotypes or treatment groups prior to data collection as non-subjective measures were used to describe observations.

Reporting for specific materials, systems and methods

We require information from authors about some types of materials, experimental systems and methods used in many studies. Here, indicate whether each material, system or method listed is relevant to your study. If you are not sure if a list item applies to your research, read the appropriate section before selecting a response.

Materials & experimental systems

n/a	Involved in the study	
☐	☒	Antibodies
☐	☒	Eukaryotic cell lines
☒	☐	Palaeontology and archaeology
☐	☒	Animals and other organisms
☒	☐	Human research participants
☒	☐	Clinical data
☒	☐	Dual use research of concern

Methods

n/a	Involved in the study	
☒	☐	ChIP-seq
☐	☒	Flow cytometry
☒	☐	MRI-based neuroimaging

Antibodies

Antibodies used	Primary antibodies used for Western blot included Sting (13647; CST, 1:1000), phospho-Sting (72971; CST, 1:1000), Tbk1 (3504; CST, 1:1000), phospho-Tbk1 (5483; CST, 1:1000), Irf3 (4302; CST, 1:1000), p65 (8242; CST, 1:1000), phospho-p65 (3033; CST, 1:1000), LC3B (MAB85582; R&D, 1:5000), Ifnar1 (ab124764; Abcam, 1:1000), Tubulin (T5168; Sigma, 1:20,000), Rab14 (15662-1-AP, Proteintech), cGAS (83623; CST 1:1000), Mavs (4983; CST, 1:1000), Sec24c (ab241336; Abcam, 1:1000), Npc1 (ab108921; Abcam, 1:1000), p-ATM (05-740; Sigma, 1:5000), FLAG (F1804; Sigma, 1:5000), FLAG (14793; CST, 1:5000), MYC (2276; CST, 1:5000), HA (3724; CST, 1:5000), Gcc2 antibody was kindly provided by Suzanne Pfeiffer (Stanford University).. Primary antibodies used for microscopy imaging included Sting (19851-1-AP; Proteintech, 1:200), GM130 (610822; BD, 1:200), TGN38 (AHP499G; Bio-Rad, 1:200), Hsp60 (sc-13115; Santa Cruz, 1:200), PDI (ab2792; Abcam, 1:200), phospho-Tbk1 (5483; CST, 1:100), phospho-Irf3 (29047; CST, 1:200), HA (3724; CST, 1:1000). Secondary antibody used included goat anti–rabbit IgG-HRP conjugate (1706515; Bio-Rad, 1:3000), goat anti–mouse IgG-HRP conjugate (1706516; Bio-Rad, 1:3000), TidyBlot Western Blot Detection Reagent:HRP (STAR209PA; Bio-Rad, 1:200), donkey anti-Rabbit IgG Alexa Fluor Plus 488 (#A-32790; ThermoFisher, 1:1000), donkey anti-Sheep IgG Alexa Fluor 594 (#A-11016; ThermoFisher, 1:1000), donkey anti-Mouse IgG Alexa Fluor 647 (#A-31571; ThermoFisher, 1:1000).
Validation	All antibodies used in this study were purchased from commercial sources and validated by the vendors (except for anti-Gcc2, which is kindly provided and validated by Suzanne Pfeiffer at Stanford University). Validation data are available on the manufacturer's websites. We further verified specificity by using isotype controls. We validated anti-Gcc2 antibody by comparing Gcc2+/+ and Gcc2-/- cells.

Eukaryotic cell lines

Policy information about cell lines

Cell line source(s)	B16F10, HEK293T, THP-1, HeLa cells were from ATCC. MEFs were generated from mouse embryos at E13.5.
Authentication	All cells were originally obtained from ATCC cell repository and not authenticated further.
Mycoplasma contamination	All cells were tested negative for mycoplasma contamination by PCR.
Commonly misidentified lines (See ICLAC register)	No commonly misidentified cell lines were used.

Animals and other organisms

Policy information about studies involving animals; ARRIVE guidelines recommended for reporting animal research

Laboratory animals	Wildtype (C57BL/6J), Rag1-/-, Ifnar1-/- ,Gcc2-/- (C57BL6/J background) and Rab14+/- mice (C57BL6/J background were used. Both sex were used at the age of 6-8 weeks unless otherwise noted in the main text or figure legends. Animal facility is level 2 barrier facility with 12h light/dark cycle and 21 degree Celsius.
Wild animals	This study did not involve wild animals.
Field-collected samples	This study did not involve field-collected samples.
Ethics oversight	All animal work are approved by the Institutional Animal Care and Use Committee at University of Texas Southwestern Medical Center.

Note that full information on the approval of the study protocol must also be provided in the manuscript.

Flow Cytometry

Plots

Confirm that:

- [x] The axis labels state the marker and fluorochrome used (e.g. CD4-FITC).
- [x] The axis scales are clearly visible. Include numbers along axes only for bottom left plot of group (a 'group' is an analysis of identical markers).
- [x] All plots are contour plots with outliers or pseudocolor plots.
- [x] A numerical value for number of cells or percentage (with statistics) is provided.

Methodology

Sample preparation	HSV-1-GFP infected cells were collected and washed with PBS. Cells were then fixed with 4% PFA and washed prior to the flow analysis.
Instrument	BD Calibur
Software	BD FACStation, FlowJo X
Cell population abundance	This study did not involve a cell sorting experiment.
Gating strategy	The non-infected cells were used as the negative control to set the gate. The same gate is applied to all samples in the same experiment. Gating strategy is presented in the main figures.

- [ ] Tick this box to confirm that a figure exemplifying the gating strategy is provided in the Supplementary Information.
